# Identification of the *SET* Family and Key Role of *ZmSET9* in Drought Tolerance in Maize (*Zea mays*)

**DOI:** 10.3390/plants15142224

**Published:** 2026-07-21

**Authors:** Huixin Zhang, Xinyu Wang, Zhengyu Wei, Xueyu Cui, Yujiao Peng, Baoqing Hu, Xiaoyu Zhang, Fulei Mo

**Affiliations:** 1 Key Laboratory of Environment Change and Resources Use in Beibu Gulf, Ministry of Education, Nanning Normal University, Nanning 530001, China; zhanghuixin_123@163.com (H.Z.);; 2 College of Life Sciences, Northeast Agricultural University, Harbin 150030, China; 3 School of Computer Science and Technology, Harbin Institute of Technology, Harbin 150001, China; 18804558224@163.com; 4 Yulin Academy of Agricultural Sciences, Yulin 537000, China; 5 School of Life Science and Technology, Harbin Institute of Technology, Harbin 150001, China

**Keywords:** drought stress, histone methylation, *ZmSET9* gene family, *Zea mays*

## Abstract

Maize (*Zea mays* L.) productivity is severely constrained by drought stress. Although the maize SET domain-containing gene family has previously been investigated, the earlier analysis was based on the incomplete B73 RefGen_v2 genome assembly, and the drought-responsive functions of individual *ZmSET* members remain largely uncharacterized. In this study, 47 *ZmSET* genes were identified using the updated B73 RefGen_v5 genome and systematically analyzed for their physicochemical properties, chromosomal distribution, gene structures, conserved motifs, and promoter cis-acting elements. The *ZmSET* family exhibited substantial evolutionary conservation, while its promoters contained numerous stress- and hormone-responsive elements. Transcriptome analysis identified *ZmSET9* as a drought-responsive gene, and RT-qPCR showed that it maintained relatively high expression throughout drought treatment. Heterologous overexpression of *ZmSET9* in *Arabidopsis thaliana* enhanced drought tolerance and supported plant growth under drought stress. Compared with wild type plants, the transgenic lines exhibited higher superoxide dismutase (SOD), catalase (CAT), and peroxidase (POD) activities and lower malondialdehyde (MDA) contents, indicating enhanced antioxidant capacity and reduced membrane lipid peroxidation. Under PEG induced osmotic stress, *AtCAT1* and *AtMYC2* were more strongly induced in the transgenic lines. Protein–protein interaction prediction and yeast two-hybrid assays further demonstrated that ZmSET9 physically interacts with FERTILIZATION-INDEPENDENT ENDOSPERM 1 (FIE1), a core component of Polycomb repressive complex 2. These findings update the genomic characterization of the maize *SET* family and suggest that *ZmSET9* contributes to drought tolerance by enhancing antioxidant defense and regulating stress-responsive gene expression.

## 1. Introduction

Plant growth, development, and environmental adaptation are regulated by genomic information and epigenetic mechanisms that modulate chromatin structure and gene expression [1,2]. Histone methylation is a major epigenetic modification involved in transcriptional regulation, genome stability, and development, and its effects depend on the modified site and methylation state [3,4]. SET domain-containing proteins are a major class of histone methyltransferases that transfer methyl groups from S-adenosyl-L-methionine to specific lysine residues and commonly mediate H3K4, H3K9, H3K27, H3K36, and H4K20 methylation [5,6]. Yeast *ScSET1* and Arabidopsis *ATX1* mainly mediate H3K4 methylation [7,8], whereas CURLY LEAF (CLF) regulates floral development by repressing *AGAMOUS* (*AG*) expression [9]. SET gene families have been identified in *Arabidopsis thaliana*, *Oryza sativa*, *Solanum lycopersicum*, and *Solanum tuberosum* [10,11,12,13]. In Arabidopsis, these proteins are classified into the E(z), ASH1, Trx, and Su(var) subfamilies and function in development, chromatin regulation, and stress responses [10,14].

SET proteins also contribute to plant adaptation to abiotic stress [15,16,17,18], and histone methylation participates in transcriptional reprogramming and stress memory under cold, salinity, and drought conditions [15]. Several *SiSET* genes in foxtail millet are induced by cold, and *SiSET14* overexpression enhances cold tolerance in yeast [19]. In tomato, *SlSET6* positively regulates salt tolerance by affecting antioxidant enzyme activity and proline accumulation [12]. In cotton, *GhSDG51* was identified as a positive regulator of salt tolerance [20]. In rice, *OsSDG721* enhances saline-alkali tolerance by promoting H3K4me3 deposition and activating stress-responsive gene expression [21]. Multiple potato *StSET* genes respond to drought and heat [11]. In Arabidopsis, *ATX1* participates in ABA-dependent and ABA-independent drought signaling through H3K4me3 regulation, while phosphatidylinositol 5-phosphate affects *ATX1*-dependent gene expression during drought stress [7,8,22]. These findings indicate conserved roles of SET proteins in abiotic stress responses, but their functions in major crops remain incompletely understood.

Maize (*Zea mays* L.) is a globally important crop whose growth and productivity are frequently constrained by drought. Drought-induced water deficit restricts cell expansion, stomatal conductance, and photosynthesis, disrupts reactive oxygen species homeostasis, and promotes membrane lipid peroxidation and cellular damage, ultimately reducing plant growth and yield. Springer et al. [23] conducted an early comparative analysis of maize and Arabidopsis SET proteins, and Qian et al. [24] identified 43 *ZmSET* genes using the B73 RefGen_v2 genome and reported differential expression of 12 members under PEG and NaCl treatments. However, the earlier reference genome was incomplete, and the functions of individual *ZmSET* genes in drought-related responses remain poorly characterized. Building on previous studies, we used the updated B73 RefGen_v5 assembly [25] to further characterize the *ZmSET* family and combined transcriptome, RT-qPCR, and functional analyses to investigate the roles of its members in drought responses. Specifically, this study aimed to identify and classify *ZmSET* genes, analyze their structural and evolutionary characteristics, screen drought-responsive candidates, evaluate the function of *ZmSET9* under drought and PEG-induced osmotic stress, and examine its interaction with FIE1, thereby providing an updated framework for the maize *SET* family and candidate genes for investigating epigenetic regulation and improving drought resilience.

## 2. Results

### 2.1. Identification and Physicochemical Characterization of the ZmSET Gene Family

Based on the maize B73 RefGen_v5 reference genome, we identified 47 members of the *ZmSET* gene family using hidden Markov model (HMM) profiling combined with BLASTP homology searching, followed by validation of the SET domain (PF00856) (Table S1). Systematic analysis of the physicochemical properties of the 47 ZmSET proteins revealed that their amino acid sequence lengths range from 192 aa (ZmSET7) to 2261 aa (ZmSET22), with corresponding molecular weights spanning 21.37 kDa to 255.55 kDa. This marked variation in sequence length and molecular mass suggests substantial functional divergence among SET family members. The isoelectric point (pI) values ranged broadly from 4.58 (ZmSET47, acidic) to 9.88 (ZmSET5, strongly basic). This wide pI range, encompassing acidic, neutral, and basic states, may facilitate the maintenance of appropriate charge states and conformational stability for individual members within their respective subcellular microenvironments. Grand average of hydropathicity (GRAVY) analysis showed that the majority of ZmSET proteins possess negative GRAVY values, indicating a predominantly hydrophilic nature.

Subcellular localization analyses predicted that approximately 81% (38 of 47) of ZmSET members localize to the nucleus, which is consistent with the established roles of SET domain-containing proteins in histone modification and transcriptional regulation. A small number of members (e.g., ZmSET7 and ZmSET28) were predicted to localize to mitochondria, plastids, or the cytoplasm. This suggests that the family may perform as-yet-unrecognized functions in the epigenetic modification of semi-autonomous organelles and inter-compartmental signal transduction; however, these non-nuclear localization predictions require experimental validation.

### 2.2. Phylogenetic Analysis of the ZmSET Gene Family

**Figure 1:**
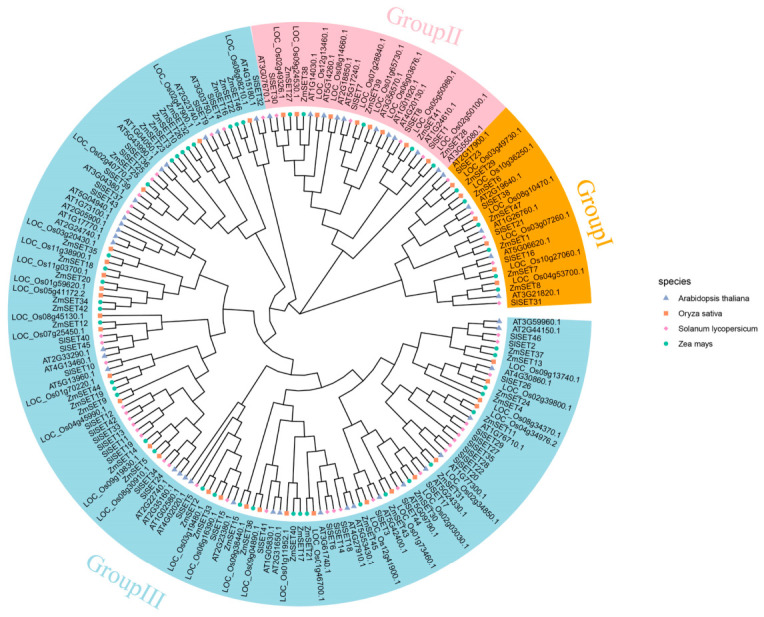
Multi-species phylogenetic analysis of the SET protein families from maize, Arabidopsis, rice, and tomato. The phylogenetic tree was constructed based on the SET protein sequences of maize (green circles), Arabidopsis (blue triangles), rice (orange squares), and tomato (purple diamonds). Based on their evolutionary relationships, these members are classified into three major clades: Group I (orange), Group II (pink), and Group III (blue).

To resolve the interspecific evolutionary relationships of the *ZmSET* gene family, we performed a multiple sequence alignment of the identified ZmSET protein sequences with their SET orthologs from *Arabidopsis thaliana*, *Oryza sativa*, and *Solanum lycopersicum*, followed by the construction of a multi-species phylogenetic tree (Figure 1). The tree topology partitioned the *SET* family members from all four species into three major clades (Group I, Group II, and Group III), with Group III containing the largest number of members. Within each clade, the ZmSET proteins largely formed tight clusters with their Poaceae orthologs from rice, indicating close evolutionary proximity, while maintaining a well-defined correspondence with the homologous sequences from Arabidopsis and tomato. This topology reflects the deep phylogenetic conservation of the *SET* gene family across the angiosperm lineage.

### 2.3. Gene Structure and Conserved Motif Analysis of the ZmSET Gene Family

**Figure 2:**
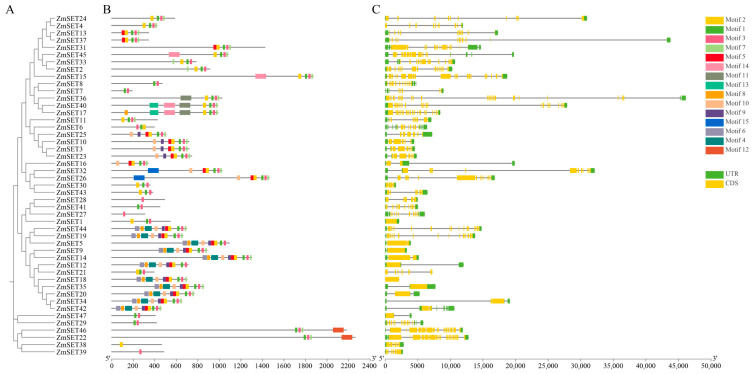
Phylogenetic tree, conserved motifs, and gene structure analysis of the *ZmSET* gene family. (**A**) Phylogenetic tree constructed based on the 47 ZmSET protein sequences. (**B**) Conserved motif composition analyzed by MEME. A total of 15 conserved motifs were identified and are represented by distinct colored boxes. (**C**) Exon–intron structures of the *ZmSET* genes. Yellow boxes indicate coding sequences (CDS), green boxes represent untranslated regions (UTRs), and black lines represent introns.

To elucidate the evolutionary relationships and structural features of the *ZmSET* gene family, we constructed a phylogenetic tree and performed an integrated analysis incorporating conserved motif composition and gene structure (Figure 2). Phylogenetic analysis resolved the 47 *ZmSET* members into multiple distinct subfamilies. MEME motif analysis identified 15 conserved motifs, with members within the same phylogenetic clade displaying highly consistent motif composition and arrangement, supporting the functional conservation of core biochemical activities within each subfamily. We further examined the exon–intron organization of all 47 *ZmSET* genes. The results revealed substantial structural diversification among family members (Table S3): exon numbers ranged from 1 (*ZmSET1* and *ZmSET18*) to 26 (*ZmSET36*), and intron numbers ranged from 0 (*ZmSET1* and *ZmSET18*) to 25 (*ZmSET36*). *ZmSET1* and *ZmSET18* possessed the simplest architecture, each consisting of a single exon with no introns, whereas *ZmSET36* exhibited the most complex structure, containing 26 exons. This structural diversity reflects the extensive gene differentiation within the *ZmSET* family over the course of evolution. Taken together, these results indicate that the *ZmSET* gene family has maintained conservation of core regulatory modules while achieving functional specialization through sequence and gene structural diversification.

### 2.4. Promoter Cis-Acting Element Analysis of the ZmSET Gene Family

**Figure 3:**
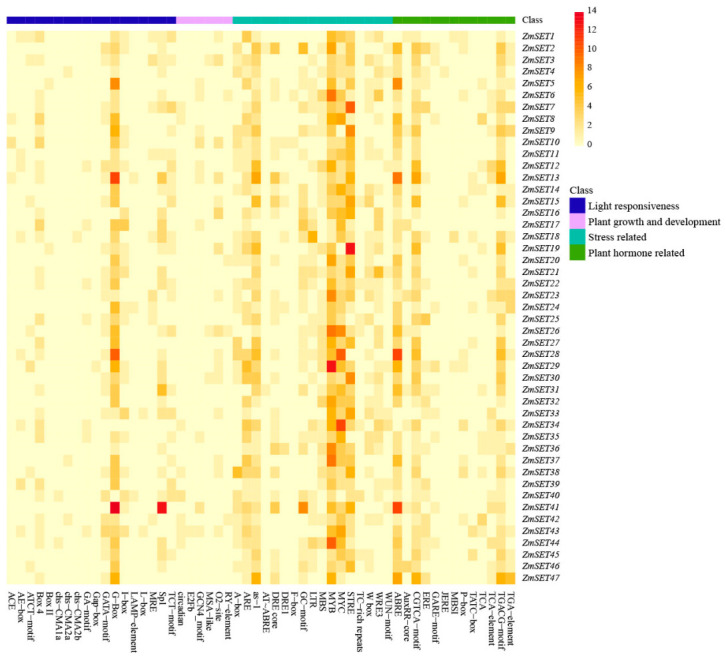
Promoter *cis*-acting element analysis of the *ZmSET* gene family. The horizontal axis represents the names of specific *cis*-acting elements, and the vertical axis indicates the *ZmSET* gene names. The *cis*-acting elements are classified into four functional categories (Class): light responsiveness, plant growth and development, environmental stress responsiveness, and phytohormone responsiveness. The color scale indicates the number of corresponding elements present within each promoter region, with colors transitioning from pale yellow (low abundance) to red (high abundance).

To investigate the potential transcriptional regulatory mechanisms governing the *ZmSET* gene family, we extracted the 2000 bp upstream sequences from the transcription start site of each of the 47 *ZmSET* genes and performed a comprehensive visualization of their *cis*-acting regulatory elements. The analysis revealed that the promoter regions of *ZmSET* genes harbor a diverse repertoire of regulatory elements, which we classified into four functional categories based on their annotated roles: environmental stress responsiveness, phytohormone responsiveness, light responsiveness, and plant growth and development (Figure 3).

Within the stress-responsive category, we found that the promoter regions of the vast majority of *ZmSET* genes are highly enriched in MYB and MYC transcription factor binding sites, ARE (anaerobic induction essential element), and STRE (stress response element) motifs, with these elements broadly distributed across the family. In the phytohormone-responsive category, abscisic acid (ABA)-responsive elements (ABRE) and methyl jasmonate (MeJA)-responsive elements (CGTCA-motif and TGACG-motif) were present at markedly higher abundance than other hormone-associated elements. This suggests that the transcriptional regulation of *ZmSET* genes is closely linked to ABA and MeJA signaling pathways and that the family may play important roles in stress hormone-mediated transcriptional reprogramming. In the light-responsive category, elements such as G-box and Box 4 were detected at high frequency in numerous members, indicating that light signaling may contribute to the regulation of a subset of *ZmSET* genes. In the plant growth and development category, elements including GCN4_motif (associated with endosperm-specific expression), O2-site (involved in zein metabolism), and the circadian regulatory motif were sporadically distributed among specific members, suggesting that particular *ZmSET* members may perform fine-tuned, spatiotemporally controlled regulatory functions in processes such as grain development, storage protein metabolism, and circadian rhythm modulation. Collectively, the functional diversity of *cis*-acting elements in the promoter regions of *ZmSET* genes suggests that the family may serve as a key integrative node coordinating environmental responses with developmental programs.

### 2.5. Chromosomal Distribution and Intra-Genomic Collinearity Analysis of the ZmSET Gene Family

**Figure 4:**
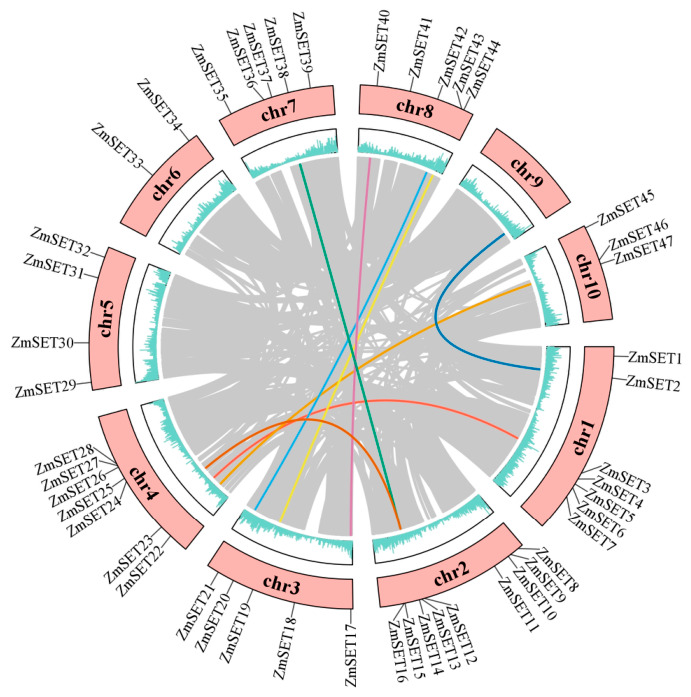
Chromosomal distribution and intra-genomic synteny analysis of the *ZmSET* gene family in maize. The Circos plot illustrates the physical mapping and collinear relationships of 47 *ZmSET* genes across the 10 maize chromosomes. The outermost circle represents the individual maize chromosomes (Chr1–Chr10) distinguished by different colors. The inner bar charts indicate the distribution density of *ZmSET* genes on each chromosome. The central colored lines denote the paralogous *ZmSET* gene pairs resulting from segmental duplications.

Using TBtools software, we generated a Circos plot (Figure 4), which revealed an uneven distribution of the 47 *ZmSET* genes across the 10 maize chromosomes. Specifically, chromosome 2 harbored the highest number of family members (nine genes, *ZmSET8* to *ZmSET16*), followed by chromosomes 1 and 4. In contrast, chromosome 6 contained only two members, and no *ZmSET* genes were localized on chromosome 9. Furthermore, intra-species collinearity analysis identified multiple paralogous *ZmSET* gene pairs across different chromosomes (Table S2). Notably, the syntenic network demonstrated that collinear relationships exist not only among *ZmSET* paralogs but also between *ZmSET* members and various non-family genes within conserved chromosomal blocks. This extensive cross-chromosomal synteny strongly indicates that large-scale segmental duplications and whole-genome duplication (WGD) events were the primary evolutionary forces driving the expansion of the *ZmSET* gene family in maize.

### 2.6. Interspecies Synteny Analysis of the ZmSET Gene Family

**Figure 5:**
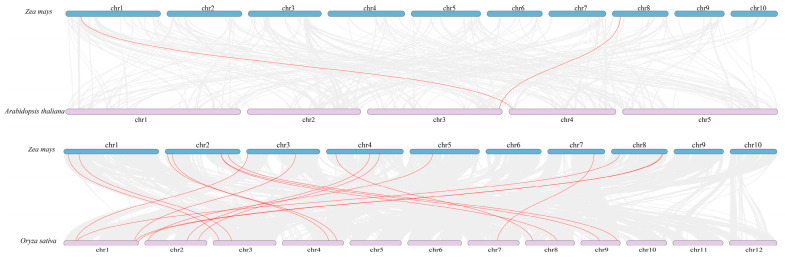
Interspecies synteny analysis of the *ZmSET* gene family with Arabidopsis and rice. The synteny maps display the collinear relationships between maize (*Zea mays*) and two representative species: Arabidopsis (*Arabidopsis thaliana*) and rice (*Oryza sativa*). Gray lines in the background indicate the collinear blocks across the entire genomes, while the red lines specifically highlight the orthologous *SET* gene pairs.

To further elucidate the interspecies syntenic relationships of the *ZmSET* gene family, we constructed cross-species synteny maps comparing maize with Arabidopsis (*Arabidopsis thaliana*) and rice (*Oryza sativa*) (Figure 5). The synteny analysis revealed that the number of orthologous *SET* gene pairs among these species exhibits significant characteristics of evolutionary divergence. Between maize and Arabidopsis, only two orthologous *SET* gene pairs (involving *ZmSET2* and *ZmSET40*) were identified at the whole-genome level. In contrast, between maize and rice (both members of the Poaceae family), we identified 17 conserved syntenic *SET* gene pairs (Table S4). Notably, these pairings exhibited both one-to-multiple (e.g., *ZmSET14* corresponding to *Os08t0400200-00* and *Os09t0362900-01*) and multiple-to-one (e.g., *ZmSET17* and *ZmSET40* corresponding to *Os01t0218800-01*) relationships. This pattern indicates that the *SET* gene family underwent substantial expansion driven by whole-genome duplication (WGD) events following the divergence of the Poaceae lineage, while still retaining robust synteny. Collectively, these results demonstrate that *ZmSET* genes share closer phylogenetic homology with their rice counterparts, strongly implying that they may exert similar biological functions.

### 2.7. Functional Annotation of ZmSET Proteins Based on GO Enrichment

**Figure 6:**
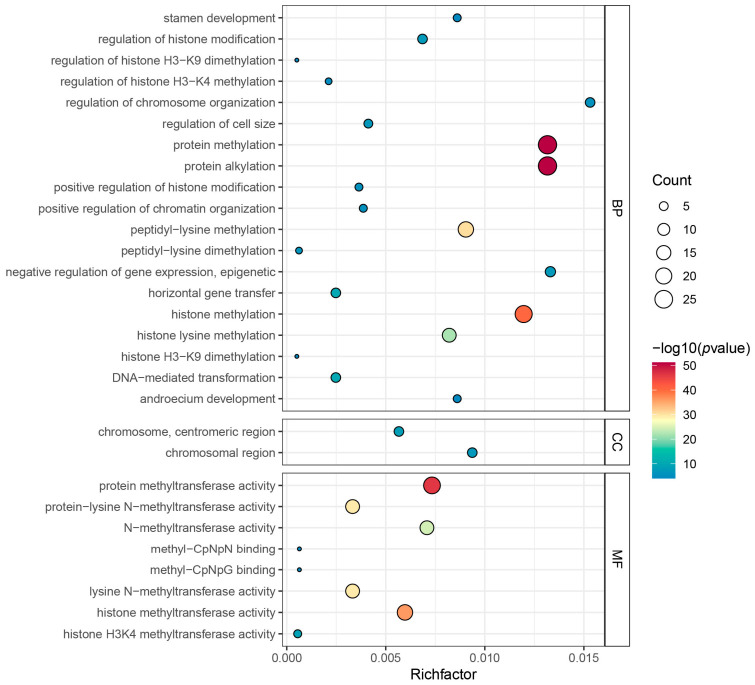
GO functional enrichment analysis of the *ZmSET* gene family. The *y*-axis lists the specific GO terms, and the *x*-axis represents the Rich factor, which reflects the proportion of enriched genes. The size of each dot corresponds to the number of genes assigned to that term. The color gradient indicates statistical significance based on the −log10 (*p*-value), with higher values representing lower *p*-values and greater significance.

To comprehensively characterize the biological functions of the *ZmSET* gene family, we performed Gene Ontology (GO) enrichment analysis. The results revealed that *ZmSET* genes were significantly enriched across all three GO categories (Figure 6): molecular function (MF), biological process (BP), and cellular component (CC).

At the molecular function level, *ZmSET* genes were predominantly enriched in protein methyltransferase activity and histone methyltransferase activity, including specific histone H3K4 methyltransferase activity; a small subset of members were additionally associated with DNA methylation-directed binding functions (e.g., methyl-CpNpG/N binding). Within the biological process category, the most prominently enriched terms included protein and histone methylation/alkylation and epigenetic regulation of gene expression. The analysis further uncovered potential roles for this family in specific plant developmental programs, with significant enrichment detected in stamen development, androecium development, and regulation of cell size. At the cellular component level, ZmSET-encoded proteins were significantly enriched in the chromosomal region and centromeric region, consistent with their established roles in epigenetic regulation.

### 2.8. Expression Profiling of the ZmSET Gene Family Under PEG-Induced Osmotic Stress

**Figure 7:**
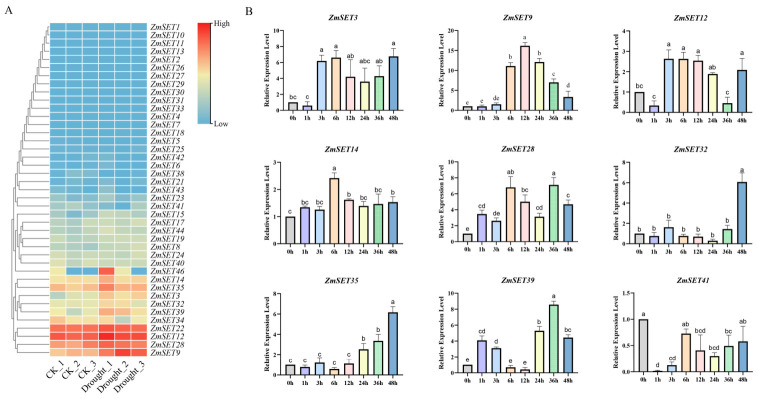
Expression patterns of the *ZmSET* gene family under drought stress. (**A**) Heatmap of *ZmSET* gene expression based on transcriptome data under drought stress. ‘CK’ indicates the well-watered control group, while ‘Drought’ represents the drought treatment group; the suffixes _1, _2, and _3 denote the three biological replicates. The color scale on the right represents the expression levels. (**B**) RT-qPCR validation of nine differentially expressed *ZmSET* genes in response to PEG-induced osmotic stress. The evaluated time points were 0, 1, 3, 6, 12, 24, 36, and 48 h. The experimental data are presented as the mean ± standard error from at least three independent biological replicates. Different letters indicate significant differences, *p* < 0.05.

To further investigate the response patterns of the *ZmSET* gene family under drought stress conditions, we systematically analyzed the expression profiles of all 47 *ZmSET* members using publicly available transcriptome datasets (SRA) from drought-treated maize samples. The results showed that drought treatment induced transcriptional changes in a substantial subset of family members: *ZmSET14*, *ZmSET35*, *ZmSET3*, *ZmSET32*, *ZmSET39*, *ZmSET12*, *ZmSET28*, and *ZmSET9* exhibited varying degrees of upregulation relative to the control, whereas *ZmSET41* was downregulated (Figure 7A).

To validate the reliability of the transcriptome data, we selected nine *ZmSET* genes that were differentially expressed under drought stress for RT-qPCR analysis (Figure 7B). The results revealed distinct temporal expression patterns among these genes over the treatment course. *ZmSET9* displayed the most pronounced response: its transcript level began to increase rapidly at 6 h post-treatment, peaked at 12 h (approximately 16-fold relative to the control), and remained elevated at 24 h and 36 h, indicating that *ZmSET9* is capable of rapid induction and sustained participation in the drought stress response. *ZmSET3* was also markedly induced, with transcript levels rising sharply to over 6-fold at 3–6 h and persisting at high levels throughout the treatment period. *ZmSET12* was significantly upregulated between 3 and 12 h (approximately 2.5-fold), followed by a gradual decline, and increased again at 48 h. *ZmSET14* reached its maximal expression at 6 h (approximately 2.4-fold) and remained relatively stable thereafter. *ZmSET28* exhibited a biphasic induction pattern, with significant upregulation at both 6 h and 36 h, reaching peak expression at 36 h (approximately 7-fold). In contrast to the above genes, *ZmSET32* and *ZmSET35* displayed late-phase response profiles: *ZmSET32* peaked at 48 h (approximately 6-fold), whereas *ZmSET35* was progressively upregulated and reached its maximum at 48 h (approximately 6-fold). *ZmSET39* was significantly induced at 24 h and 36 h, peaking at 36 h (approximately 8.5-fold) before declining slightly. In contrast, *ZmSET41* exhibited an overall downward trend following drought treatment, reaching its minimum at 1 h; despite a modest recovery at later time points, its transcript levels remained consistently below those of the control.

Collectively, the majority of the examined *ZmSET* genes were significantly induced by drought stress, with *ZmSET9*, *ZmSET28*, and *ZmSET39* showing the greatest magnitudes of upregulation, whereas ZmSET41 displayed a sustained decline in expression. This pattern of differential expression suggests that individual *ZmSET* family members perform distinct functions in the maize drought stress response. Given its most pronounced and sustained induction profile, we selected *ZmSET9* as the core candidate for further functional characterization.

### 2.9. ZmSET9 Overexpression Enhances Drought Tolerance in Arabidopsis

**Figure 8:**
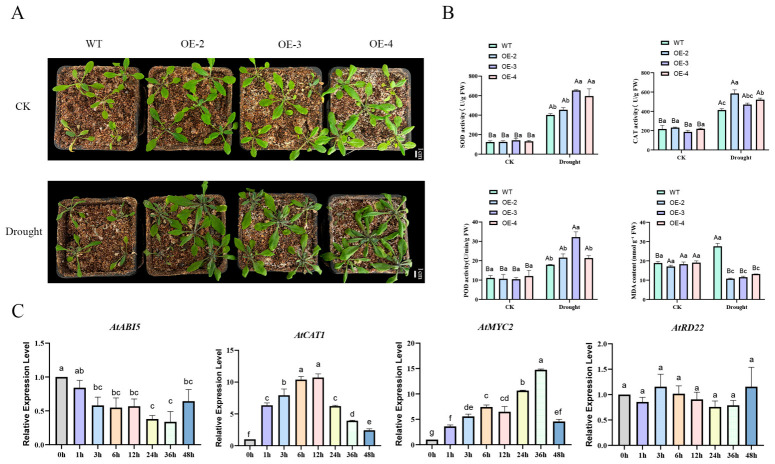
Responses of *ZmSET9*-overexpressing Arabidopsis plants to drought stress. (**A**) Representative phenotypes of wild-type (WT) and *ZmSET9*-overexpressing lines (OE-2, OE-3, and OE-4) under well-watered control conditions (CK) and after withholding water for 14 d. (**B**) SOD, CAT, and POD activities, as well as MDA content, in WT and OE lines under CK and drought conditions. (**C**) Relative expression levels of *AtABI5*, *AtCAT1*, *AtMYC2*, and *AtRD22* at 0, 1, 3, 6, 12, 24, 36, and 48 h after PEG-induced osmotic stress treatment. Data are presented as mean ± standard error (SE), and all experiments were performed with at least three independent biological replicates. In (**B**), different lowercase letters indicate significant differences among genotypes under the same treatment, whereas different uppercase letters indicate significant differences between CK and drought treatments within the same genotype. In (**C**), different lowercase letters indicate significant differences among treatment time points (*p* < 0.05).

Based on the consistent results of transcriptome and RT-qPCR analyses, together with its rapid, strong, and sustained induction under drought stress, *ZmSET9* was selected for further functional characterization. To investigate the role of *ZmSET9* in plant drought responses, *ZmSET9* was heterologously expressed in Arabidopsis, and stable homozygous T_3_ transgenic lines were obtained. Three overexpression lines, OE-2, OE-3, and OE-4, were selected for subsequent analyses (Figure S1). As shown in Figure 8A, no obvious phenotypic differences were observed between wild-type (WT) plants and the OE lines under well-watered conditions. After water was withheld for 14 d, WT plants exhibited severe wilting and growth inhibition, whereas the OE lines maintained better growth and retained more green leaves, indicating that *ZmSET9* overexpression enhanced drought tolerance in Arabidopsis.

Physiological analyses showed that Superoxide dismutase (SOD), catalase (CAT), and peroxidase (POD) activities and malondialdehyde (MDA) content differed only slightly among the genotypes under well-watered conditions. Following drought treatment, the OE lines generally exhibited higher SOD, CAT, and POD activities than WT plants, with OE-3 showing the most pronounced response. Meanwhile, MDA content was significantly lower in the OE lines than in WT plants (Figure 8B). These results indicate that *ZmSET9* overexpression strengthens antioxidant defenses and alleviates drought-induced membrane lipid peroxidation.

The expression patterns of *AtABI5*, *AtCAT1*, *AtMYC2*, and *AtRD22* were further examined during 0–48 h of PEG-induced osmotic stress (Figure 8C). *AtABI5* expression generally decreased before partially recovering at later time points. *AtCAT1* was significantly induced and reached its peak at 12 h, with an expression level more than 10-fold higher than that at 0 h. *AtMYC2* was also strongly induced and peaked at 36 h, reaching approximately 15-fold the level observed at 0 h. By contrast, *AtRD22* showed only minor fluctuations, with no significant differences among the examined time points. Collectively, these results suggest that *ZmSET9* overexpression may improve plant adaptation to drought stress by enhancing antioxidant capacity and modulating the expression of stress-responsive genes such as *AtCAT1* and *AtMYC2*.

### 2.10. Prediction and Yeast Two-Hybrid Validation of ZmSET9 Interacting Proteins

**Figure 9:**
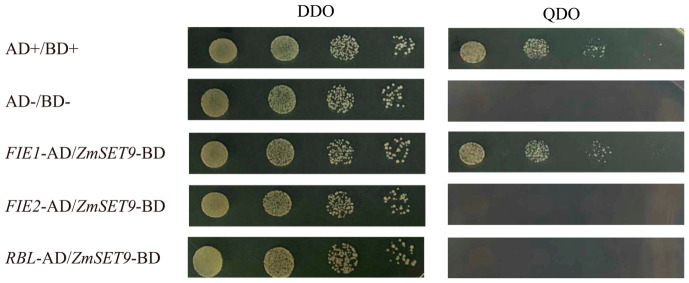
Yeast two-hybrid validation of candidate ZmSET9-interacting proteins. Y2H validation of interactions between ZmSET9 and candidate proteins. The bait vector (pGBKT7-*ZmSET9*) was co-transformed with the respective prey vectors (pGADT7-*FIE1*, pGADT7-*FIE2*, and pGADT7-*RBL*) into Y2HGold yeast competent cells. The transformed cells were plated onto double dropout (DDO, SD/-Trp/-Leu) and quadruple dropout (QDO, SD/-Trp/-Leu/-His/-Ade) selective media and incubated at 30 °C for 3–5 days to observe yeast growth.

Given that the *ZmSET9* gene exhibited the most significant expression response under drought stress, we further predicted its protein–protein interaction (PPI) network using the STRING database (Figure S2). Based on the interaction scores, the top three candidate interacting proteins—FIE1 (fertilization-independent endosperm 1), FIE2 (fertilization-independent endosperm 2), and RBL (RbBP5-like)—were selected for yeast two-hybrid (Y2H) experimental validation (Figure 9). The results demonstrated a clear physical interaction between the ZmSET9 protein and FIE1, a core subunit of the Polycomb Repressive Complex 2 (PRC2). This finding strongly suggests that ZmSET9 may collaborate with PRC2 components such as FIE1 to participate in the regulation of histone H3 lysine 27 trimethylation (H3K27me3) deposition, thereby playing a critical role in the epigenetic regulation of the drought stress response in maize.

## 3. Discussion

### 3.1. Genome-Wide Identification and Evolutionary Expansion of the ZmSET Gene Family

With the continuous advancement of high-throughput sequencing technologies and the progressive improvement in genome assembly quality, the re-identification and in-depth characterization of gene families based on higher-quality reference genomes have become a fundamental component of functional genomics research [25]. In the present study, we identified 47 *ZmSET* gene family members at the whole-genome level using the latest maize B73 RefGen_v5 reference assembly, representing an increase of four genes over the 43 members reported by Qian et al. (2014) based on the RefGen_v2 assembly [24]. The recovery of these additional members can be attributed to the markedly improved assembly quality of RefGen_v5, which has resolved numerous gaps and corrected misassemblies present in RefGen_v2 [25], thereby enabling the accurate annotation of *SET* genes that were previously missed owing to assembly incompleteness. This finding also suggests that further members of this family may yet be discovered as reference genome assemblies continue to improve—for example, with the advent of telomere-to-telomere (T2T) gap-free genomes—and warrants continued attention in future studies.

Intra-genomic synteny analysis revealed extensive segmental duplication relationships among ZmSET family members, indicating that whole-genome duplication (WGD) events have served as the primary driving force underlying family expansion. Cross-species synteny analysis provided direct evidence in support of this conclusion: 17 syntenic orthologous *SET* gene pairs were identified between maize and rice, whereas only two such pairs were detected between maize and Arabidopsis. This disparity demonstrates that the *SET* gene family has been substantially retained during Poaceae evolution while undergoing extensive gene loss in the dicot lineage. This finding is consistent with the early observation by Springer et al. (2003) that the *SET* gene complements of maize and Arabidopsis differ markedly [23].

### 3.2. Potential Regulatory Mechanisms Revealed by Promoter Cis-Acting Element Analysis

*Cis*-acting elements within promoter regions are key determinants of gene transcriptional regulation, and their composition and arrangement directly govern the spatiotemporal expression patterns and stress-responsive capacity of genes [26]. Our systematic analysis of the promoter regions of 47 *ZmSET* genes revealed that this family is significantly enriched in four major categories of *cis*-acting elements, with stress-responsive and phytohormone-responsive elements showing particularly prominent abundance and density. This finding is consistent with previous observations for the *SET* gene family in wheat [27].

Within the stress-responsive category, MYB and MYC transcription factor binding sites and ARE (anaerobic induction essential element) were detected at high frequency in the promoters of the vast majority of *ZmSET* members. MYB and MYC represent two of the largest transcription factor families in plants and have been demonstrated to participate extensively in the transcriptional regulatory networks governing responses to drought, high salinity, and low temperature [28,29]. The widespread distribution of MYB/MYC elements in *ZmSET* promoters suggests that this family may be subject to coordinated regulation by multiple MYB- and MYC-type transcription factors, thereby contributing to rapid stress signal transduction and transcriptional reprogramming.

Within the phytohormone-responsive category, the abundance of ABA-responsive elements (ABRE) and MeJA-responsive elements (CGTCA-motif and TGACG-motif) was markedly higher than that of other hormone-associated elements. ABA is the central hormonal signal mediating plant responses to drought stress; under water-deficit conditions, rapid ABA accumulation activates AREB/ABF transcription factors, which in turn trigger the expression of a large suite of downstream stress-responsive genes [30]. MeJA is likewise a key hormonal regulator of plant defense and stress responses and engages in extensive crosstalk with the ABA signaling pathway [31]. The enrichment of ABRE and MeJA-responsive elements in *ZmSET* promoters provides cis-regulatory evidence supporting the potential involvement of *ZmSETs* in ABA- and JA-mediated drought stress signaling.

### 3.3. Expression Patterns of the ZmSET Gene Family Under Drought Stress and Preliminary Screening of Candidate Genes

In this study, we integrated transcriptome data with RT-qPCR experiments to systematically characterize the expression response patterns of the *ZmSET* gene family under drought stress. Transcriptome analysis revealed that eight *ZmSET* members (including *ZmSET9*, *ZmSET14*, and *ZmSET35*) were significantly upregulated under drought treatment, whereas *ZmSET41* was downregulated. Subsequent RT-qPCR experiments not only validated the reliability of the transcriptome data but also resolved the temporal dynamics of these genes over the stress time course. Although the overall trends of the nine selected genes were consistent between the two approaches, discrepancies at individual time points were observed, which is an expected outcome attributable to the inherent differences between the two techniques in detection principle, dynamic range, and quantification accuracy. By leveraging the complementary strengths of both methods, we mitigated the risk of biased conclusions that might arise from reliance on a single approach.

*ZmSET9* exhibited the most pronounced drought-responsive induction profile: its transcript level increased rapidly at 6 h post-treatment (early response), peaked at 12 h (approximately 16-fold relative to the control), and remained substantially elevated at 24–36 h. In addition, *ZmSET32* and *ZmSET35* displayed predominantly late-phase responses (peaking at 48 h), whereas *ZmSET41* was persistently downregulated throughout the treatment. This pattern of divergent temporal expression—encompassing early induction, late induction, and sustained suppression among members of the same gene family—has similarly been observed in the *SET* gene families of Arabidopsis, rice, and tomato [10,12,13], suggesting that individual *SET* members may perform temporally distinct roles across the drought response continuum.

Moreover, phenotypic evaluation of *ZmSET9*-overexpressing Arabidopsis plants provides preliminary functional evidence for the involvement of *ZmSET9* in drought responses. Under drought stress, the OE lines maintained better growth than WT plants and exhibited higher SOD, CAT, and POD activities together with lower MDA accumulation. Drought stress commonly disrupts cellular reactive oxygen species (ROS) homeostasis, leading to excessive ROS accumulation and oxidative damage [32]. SOD converts superoxide radicals (O_2_^−^) into H_2_O_2_, whereas CAT and POD further remove H_2_O_2_. Therefore, the coordinated increases in these antioxidant enzyme activities are consistent with an enhanced ROS-scavenging capacity [33]. In contrast, MDA is a product of polyunsaturated fatty acid peroxidation and is widely used as an indicator of oxidative membrane damage; thus, its lower accumulation indicates reduced membrane lipid peroxidation [34]. A relevant example has also been reported for another SET family member in tomato, where silencing of SlSET6 reduced SOD, CAT, and POD activities and increased MDA accumulation under salt stress [12]. Together with the PEG-induced changes in *AtCAT1* and *AtMYC2* expression, these results support an association between ZmSET9 overexpression, enhanced antioxidant defense, and reduced membrane damage, which may contribute to the improved drought performance of the transgenic plants. However, because ROS levels and the direct regulation of antioxidant-related genes by *ZmSET9* were not examined, these findings do not establish a direct regulatory mechanism and require further validation in maize.

### 3.4. Functional Implications of the ZmSET9–FIE1 Interaction and Future Research Perspectives

In this study, through STRING database-based protein–protein interaction network prediction combined with Y2H experimental validation, we demonstrated that ZmSET9 physically interacts with FIE1. FIE1 is a core subunit of the Polycomb Repressive Complex 2 (PRC2) and has been shown to be essential for PRC2-mediated H3K27me3 deposition in both Arabidopsis and maize [35,36]. In maize, FIE1 performs a critical function in endosperm development and the regulation of genomic imprinting [36]. The PRC2 complex in plants consists of four core subunits: E(z), which harbors the SET domain and catalyzes H3K27 trimethylation; Su(z)12; FIE (also known as ESC); and MSI [37]. Within this complex, E(z) serves as the scaffold protein mediating complex assembly, whereas FIE determines the targeting specificity of PRC2 [38].

**Figure 10:**
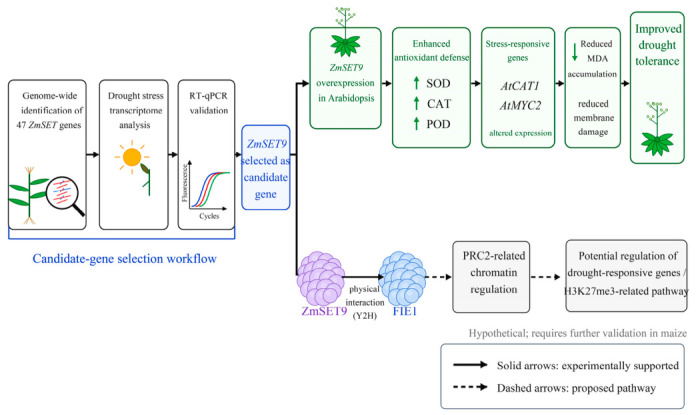
Proposed working model of *ZmSET9* involvement in plant drought responses. Following genome-wide identification of 47 *ZmSET* genes, drought-stress transcriptome analysis and RT-qPCR validation identified *ZmSET9* as a candidate gene for further functional characterization. Heterologous overexpression of *ZmSET9* in *Arabidopsis* was associated with increased SOD, CAT, and POD activities, altered expression of the stress-responsive genes *AtCAT1* and *AtMYC2*, reduced MDA accumulation and membrane damage, and improved drought tolerance. Yeast two-hybrid analysis demonstrated a physical interaction between ZmSET9 and FIE1. This interaction suggests a potential association of ZmSET9 with PRC2-related chromatin regulation and H3K27me3-dependent regulation of drought-responsive genes. The up and down arrows indicate increased antioxidant enzyme activities (SOD, CAT, POD) and decreased MDA accumulation, respectively. Solid arrows indicate relationships supported by the present experimental results, whereas dashed arrows indicate proposed regulatory relationships that require further validation in maize.

Based on the expression, physiological, transcriptional, and protein-interaction results, we propose a preliminary working model for the involvement of ZmSET9 in plant drought responses (Figure 10). Following genome-wide identification of the *ZmSET* family, drought-stress transcriptome analysis and RT-qPCR validation identified *ZmSET9* as a candidate gene for further functional characterization. Heterologous overexpression of *ZmSET9* in *Arabidopsis* was associated with increased SOD, CAT, and POD activities, altered expression of the stress-responsive genes *AtCAT1* and *AtMYC2*, reduced MDA accumulation and membrane damage, and improved drought tolerance. In parallel, the experimentally detected interaction between ZmSET9 and FIE1 suggests that ZmSET9 may be associated with PRC2-related chromatin regulation and the H3K27me3-dependent regulation of drought-responsive genes. In Figure 10, solid arrows represent relationships supported by the present experimental results, whereas dashed arrows indicate proposed regulatory relationships that remain to be validated.

However, yeast two-hybrid analysis alone is insufficient to establish that ZmSET9 participates in the PRC2 complex or that the ZmSET9–FIE1 interaction regulates H3K27me3 deposition. Additional interaction assays, including bimolecular fluorescence complementation, luciferase complementation imaging, and co-immunoprecipitation, are required to confirm this interaction in plant cells. Analyses of H3K27me3 levels and downstream target genes will also be necessary to determine whether ZmSET9 participates in PRC2-related chromatin regulation. Furthermore, because the present functional evidence was obtained primarily from heterologous overexpression in *Arabidopsis*, *ZmSET9* overexpression and knockout lines in maize will be required to establish its endogenous function and regulatory mechanism during maize drought responses.

## 4. Materials and Methods

### 4.1. Plant Material and Drought Stress Treatment

Seeds of the maize inbred line B73 were surface-sterilized and germinated in darkness at 37 °C for 24 h following immersion in distilled water. Germinated seedlings were subsequently transferred to pots containing a 1:1 (*v*/*v*) perlite–soil mixture and cultivated in a controlled growth chamber under a 16 h/8 h light/dark photoperiod with corresponding temperatures of 25 °C/22 °C (day/night). At the three-leaf stage, drought stress was imposed by irrigating seedlings with a 12% (*w*/*v*) PEG6000 solution (Beyotime, Shanghai, China) [39]. Leaf tissue was harvested at eight time points post-treatment (0, 1, 3, 6, 12, 24, 36, and 48 h), with three independent biological replicates collected per time point (~0.1 g each). All samples were snap-frozen in liquid nitrogen immediately upon collection and stored at −80 °C until further processing.

### 4.2. Identification of the ZmSET Gene Family

The B73 reference genome (Zm-B73-REFERENCE-NAM-5.0), along with its corresponding protein sequences and genome annotation files, was retrieved from the Phytozome database (https://phytozome-next.jgi.doe.gov/, accessed on 6 March 2026). To identify SET domain-containing proteins, the Hidden Markov Model (HMM) profile of the SET domain (PF00856) was downloaded from the Pfam database and employed as a query against the maize proteome using HMMER v3.3 with default parameters [40]. Concurrently, characterized SET protein sequences from Oryza sativa were used as queries in BLASTP (v2.15.0) searches against the maize protein dataset, and hits from both approaches were merged into a non-redundant candidate list. Each candidate was subsequently validated for the presence of a complete SET domain through SMART (http://smart.embl.de/, accessed on 10 March 2026), the NCBI Conserved Domain Database (https://www.ncbi.nlm.nih.gov/cdd/, accessed on 10 March 2026), and Pfam (http://pfam.xfam.org/, accessed on 10 March 2026). Sequences lacking an intact SET domain were discarded, yielding the final set of 47 *ZmSET* genes.

### 4.3. Physicochemical Property and Subcellular Localization Analysis

The ExPASy ProtParam tool (https://web.expasy.org/protparam/, accessed on 10 March 2026) was employed to compute physicochemical parameters of the deduced ZmSET proteins, including amino acid length, molecular weight (MW), theoretical isoelectric point (pI), and grand average of hydropathicity (GRAVY). Subcellular localization of each ZmSET protein was predicted using the DeepLoc-2.0 online server (https://services.healthtech.dtu.dk/services/DeepLoc-2.0/, accessed on 10 March 2026) with default settings.

### 4.4. Conserved Motif, Gene Structure, and Promoter Analysis of ZmSET

Multiple sequence alignment of ZmSET protein sequences was performed using ClustalW implemented in MEGAX, and a phylogenetic tree was constructed using the Neighbor-Joining (NJ) method [41]. Conserved protein motifs were identified using MEME Suite v5.59 (https://meme-suite.org/meme/tools/meme; accessed on 10 October 2025), with the maximum number of motifs set to 15 and motif lengths ranging from 6 to 100 amino acid residues. Exon–intron structure information was obtained from the maize genome GFF annotation file, and gene structures were visualized together with the phylogenetic tree using TBtools V2.467 [42].

Promoter sequences of the *ZmSET* gene family were extracted based on the maize genome sequence and corresponding annotation files. The obtained sequences were subsequently submitted to the PlantCARE (https://bioinformatics.psb.ugent.be/webtools/plantcare/html/, accessed on 10 October 2025) online database for *cis*-acting regulatory element analysis. The identified *cis*-acting elements were then classified and visualized using R v4.3.1.

### 4.5. Chromosomal Localization and Evolutionary Synteny Analysis

The physical positions of *ZmSET* genes on maize chromosomes were extracted from the genome annotation file and visualized using the Advanced Circos function implemented in TBtools to analyze intra-genomic syntenic relationships. For interspecies synteny analysis, genome sequences and corresponding annotation files of *Oryza sativa* and *Arabidopsis thaliana* were downloaded from the Phytozome database. Subsequently, syntenic relationships between maize and the two species were analyzed and visualized using the One Step MCScanX module in TBtools V2.467.

### 4.6. Transcriptome Analysis of the ZmSET Gene Family

Publicly available Transcriptome datasets (accession number: PRJNA952945) were downloaded from the NCBI Sequence Read Archive (https://www.ncbi.nlm.nih.gov/; accessed on 10 April 2026). Clean reads were analyzed using Salmon v1.10.3, and transcript abundance values were calculated as Transcripts Per Million (TPM) [43]. Expression profiles of all 47 *ZmSET* genes under drought stress and control conditions were extracted, and hierarchical clustering analysis was subsequently performed to generate a heatmap using the heatmap module in TBtools.

### 4.7. GO Annotation and Functional Enrichment Analysis of ZmSET Gene Family

Gene Ontology (GO) annotation of maize protein sequences was obtained using EggNOG-mapper v5 (http://eggnog5.embl.de/#/app/home, accessed on 15 April 2026). GO enrichment analysis of the *ZmSET* gene family across biological process (BP), cellular component (CC), and molecular function (MF) was conducted with clusterProfiler v4.0 in R [44]. Stress-responsive GO terms passing the significance threshold were visualized as a dot plot via ggplot2 v3.5.1 to illustrate the functional repertoire of the *ZmSET* gene family.

### 4.8. Quantitative Real-Time PCR (RT-qPCR) and Data Analysis

For RT-qPCR confirmation, leaf total RNA was isolated with TransZol Up Plus RNA Kit (TransGen Biotech, Beijing, China) and subsequently reverse-transcribed using the TransScript One-Step gDNA Removal and cDNA Synthesis SuperMix (TransGen Biotech, Beijing, China). Oligonucleotide primers targeting nine ZmSET genes and the reference gene *ZmActin* [45] were designed with NCBI Primer-BLAST (https://www.ncbi.nlm.nih.gov/tools/primer-blast/, accessed on 25 April 2026) and are listed in Table S5. Quantitative PCR was performed with TransStart Top Green qPCR SuperMix (TransGen Biotech, Beijing, China) on a QuantStudio 3 system, following the thermal cycling conditions described by Mo et al. [46]. Fold changes in gene expression relative to *ZmActin* were calculated via the 2^−ΔΔCt^ algorithm [47]. All assays included three biological replicates and three technical replicates per sample. Statistical computation and figure generation were accomplished using SPSS v26.0 and GraphPad Prism 9.0, respectively.

### 4.9. Generation of ZmSET9-Overexpressing Arabidopsis and Stress Treatments

The full-length coding sequence of *ZmSET9* was cloned into pCAMBIA1300-HA and introduced into *Agrobacterium tumefaciens* GV3101. *Arabidopsis thaliana* Col-0 plants were transformed using the floral dip method. Following hygromycin selection and molecular verification (Supplementary Figure S1, Table S5), homozygous T_3_ lines OE-2, OE-3, and OE-4 were selected for further analyses. WT and OE plants were grown at 22 °C under a 16 h light/8 h dark photoperiod. Drought stress was imposed by withholding water for 14 d, while control plants were watered normally. Plant phenotypes were recorded, and leaves were collected for physiological measurements. For short-term osmotic stress, a separate batch of plants was treated with 20% PEG6000, and leaves were sampled at 0, 1, 3, 6, 12, 24, 36, and 48 h. The expression levels of *AtABI5*, *AtCAT1*, *AtMYC2*, and *AtRD22* were determined by RT-qPCR using *AtActin* as the reference gene and calculated using the 2^−ΔΔCt^ method. Primer sequences are listed in Supplementary Table S5.

### 4.10. Antioxidant Enzyme Activities and MDA Content

SOD, CAT, and POD activities and MDA content were measured in leaves of WT and OE plants under control and drought conditions using commercial assay kits according to the manufacturer’s instructions. Values were normalized to fresh weight. All experiments included at least three independent biological replicates.

### 4.11. Yeast Two-Hybrid Screening of ZmSET9

The full-length coding sequence of *ZmSET9* was amplified and ligated into the pGBKT7 vector to construct the bait plasmid pGBKT7-*ZmSET9*, while the coding sequences of *FIE1*, *FIE2*, and *RBL* were individually inserted into pGADT7 to generate the prey plasmids pGADT7-*FIE1*, pGADT7-*FIE2*, pGADT7-*RBL*, respectively (primers listed in Table S5). Each prey construct was co-transformed with the bait plasmid into Y2HGold yeast competent cells (Coolaber, Beijing, China) using the PEG/LiAc-mediated method [48]. Co-transformed cells were initially plated on SD/-Trp/-Leu (double dropout, DDO) medium and incubated at 30 °C for 2 d. Single colonies were subsequently streaked onto SD/-Trp/-Leu/-His/-Ade (quadruple dropout, QDO) selective medium and cultured at 30 °C for an additional 3–5 d to assess protein–protein interaction. The combination of pGBKT7-*53* + pGADT7-*T* served as the positive control, while pGBKT7-*Lam* + pGADT7-*T* was used as the negative control. Growth of co-transformed yeast cells on QDO medium was scored as positive, thereby confirming direct physical interactions between ZmSET9 and FIE1.

## 5. Conclusions

Based on the maize B73 RefGen_v5 reference genome, a total of 47 *ZmSET* family members were identified in this study. Chromosomal distribution and synteny analyses showed that these genes are unevenly distributed across the 10 maize chromosomes and suggested that whole-genome duplication (WGD) may have been the major driving force underlying the expansion of this gene family. Cross-species comparisons further indicated that *SET* genes were largely retained in Poaceae, whereas extensive gene loss occurred in dicotyledonous species. Members within the same phylogenetic clade exhibited highly conserved motif composition and arrangement. Promoter analysis revealed that the upstream regions of *ZmSET* genes are enriched in stress- and hormone-responsive cis-acting elements, while GO enrichment analysis showed that these genes are mainly associated with histone methyltransferase activity, chromosomal regions, and stamen development. Integration of transcriptome and qRT-PCR analyses showed that *ZmSET9* was strongly responsive to drought stress. Overexpression of *ZmSET9* enhanced drought tolerance in Arabidopsis, as evidenced by improved plant growth under drought stress, increased activities of SOD, CAT, and POD, and reduced MDA content. In addition, *AtCAT1* and *AtMYC2* were significantly induced during PEG-induced osmotic stress, suggesting that *ZmSET9* may contribute to drought adaptation by enhancing antioxidant defense and modulating the expression of stress-responsive genes. Yeast two-hybrid assays further confirmed that ZmSET9 interacts with FIE1, a core component of the PRC2 complex. In summary, this study provides new insights into the evolutionary characteristics and stress-responsive functions of the maize *SET* gene family and suggests that *ZmSET9* is a promising candidate gene for the molecular improvement of drought tolerance in maize.

## Data Availability

The transcriptome datasets analyzed in this study are publicly available in the NCBI Sequence Read Archive (SRA) and BioProject databases under accession numbers PRJNA952945.

## Supplementary Materials

The following supporting information can be downloaded at: https://www.mdpi.com/article/10.3390/plants15142224/s1, Table S1: Analysis of physicochemical properties of *ZmSET* gene family; Table S2: Collinearity Analysis data; Table S3: The numbers of introns and exons in the *ZmSET* gene family; Table S4: Synteny analyses data; Table S5: Primer list; Figure S1: PCR identification of transgenic Arabidopsis thaliana lines; Figure S2: Protein–protein interaction network prediction of ZmSET9.
