# Supplementary material for: Identification of the SET Family and Key Role of ZmSET9 in Drought Tolerance in Maize (Zea mays)
Source: Plants (Basel). 2026 Jul 21;15(14):2224. doi: 10.3390/plants15142224 (PMC13416610; doi:10.3390/plants15142224)
Supplement: Supplementary file 1 [file plants-15-02224-s001.zip › plants-4439495-supplementary.pdf]

Table. S1. Analysis of physicochemical properties of *ZmSET* gene family

Gene ID	Protein ID	Protein Length	CDS Length (nt)	Molecular Weight (Da)	Isoelectric Point (pI)	Hydrophobicity (GRAVY)	Predicted localization
Zm00001eb005310_P001	*ZmSET1*	544	1635	58928.53	7.15	-0.17	Cytoplasm
Zm00001eb014730_P001	*ZmSET2*	912	2739	102522.35	8.56	-0.68	Nucleus
Zm00001eb038540_P001	*ZmSET3*	720	2163	79348.44	5.81	-0.45	Nucleus
Zm00001eb041060_P001	*ZmSET4*	421	1266	47414.06	9.02	-0.59	Nucleus
Zm00001eb042640_P001	*ZmSET5*	1093	3282	119368.81	9.88	-0.56	Nucleus
Zm00001eb044040_P001	*ZmSET6*	396	1191	44480.73	8.43	-0.1	Nucleus
Zm00001eb048280_P001	*ZmSET7*	192	579	21370.22	4.75	0.02	Mitochondrion
Zm00001eb069580_P001	*ZmSET8*	469	1410	51995.12	4.7	-0.24	Cytoplasm
Zm00001eb075120_P001	*ZmSET9*	886	2661	97154.46	6.81	-0.63	Nucleus
Zm00001eb076530_P001	*ZmSET10*	718	2157	78629.65	6.33	-0.53	Nucleus
Zm00001eb082920_P001	*ZmSET11*	425	1278	47367.44	5.07	-0.52	Nucleus
Zm00001eb096860_P001	*ZmSET12*	711	2136	77814.27	5.37	-0.25	Nucleus
Zm00001eb097830_P001	*ZmSET13*	343	1032	39786.04	7.47	-0.58	Nucleus
Zm00001eb098590_P001	*ZmSET14*	1301	3906	139457.1	6.09	-0.53	Nucleus
Zm00001eb104540_P001	*ZmSET15*	1872	5619	206527.34	8.8	-0.59	Nucleus
Zm00001eb104980_P001	*ZmSET16*	339	1020	35641.32	7.42	0	Mitochondrion
Zm00001eb119830_P001	*ZmSET17*	986	2961	109920.71	7.46	-0.43	Nucleus
Zm00001eb132780_P001	*ZmSET18*	699	2100	77153.24	7.38	-0.54	Nucleus
Zm00001eb142610_P001	*ZmSET19*	662	1989	73159.44	8.53	-0.56	Nucleus
Zm00001eb150160_P001	*ZmSET20*	766	2301	83841.86	5.58	-0.45	Nucleus
Zm00001eb158060_P001	*ZmSET21*	394	1185	44114.62	9.05	-0.59	Nucleus
Zm00001eb171790_P001	*ZmSET22*	2261	6786	255549.56	6.4	-0.75	Nucleus
Zm00001eb175610_P001	*ZmSET23*	743	2232	82186.56	5.62	-0.49	Nucleus
Zm00001eb184480_P001	*ZmSET24*	585	1758	65028.14	8.73	-0.3	Nucleus
Zm00001eb185000_P001	*ZmSET25*	503	1512	56261.12	6.05	-0.51	Nucleus
Zm00001eb188560_P001	*ZmSET26*	1463	4392	164267.07	6.57	-0.46	Nucleus
Zm00001eb1892	*ZmSET27*	308	927	35177.69	5.82	-0.39	Nucleus

Gene ID	Protein ID	Protein Length	CDS Length (nt)	Molecular Weight (Da)	Isoelectric Point (pI)	Hydrophobicity (GRAVY)	Predicted localization
10_P001							
Zm00001eb190260_P001	*ZmSET28*	491	1476	53821.41	8.21	0.02	Plastid
Zm00001eb216690_P001	*ZmSET29*	416	1251	47042.89	8.48	-0.18	Nucleus
Zm00001eb230520_P001	*ZmSET30*	356	1071	39004.19	9.07	-0.42	Nucleus
Zm00001eb243610_P001	*ZmSET31*	1423	4272	154844.33	6.19	-0.64	Nucleus
Zm00001eb252300_P001	*ZmSET32*	1025	3078	114895.75	5.92	-0.47	Nucleus
Zm00001eb271490_P001	*ZmSET33*	785	2358	88349.67	8.82	-0.8	Nucleus
Zm00001eb291310_P001	*ZmSET34*	652	1959	71946.61	7.93	-0.5	Nucleus
Zm00001eb303540_P001	*ZmSET35*	856	2571	93442.84	8.56	-0.51	Nucleus
Zm00001eb308480_P001	*ZmSET36*	1025	3078	116126.96	7.26	-0.53	Nucleus
Zm00001eb309540_P001	*ZmSET37*	342	1029	39502.54	6.6	-0.57	Nucleus
Zm00001eb312420_P001	*ZmSET38*	463	1392	51275.63	5.06	-0.2	Plastid
Zm00001eb320090_P001	*ZmSET39*	483	1452	54094	5.3	-0.23	Cytoplasm
Zm00001eb338670_P001	*ZmSET40*	985	2958	110099.93	8.31	-0.47	Nucleus
Zm00001eb345040_P001	*ZmSET41*	448	1347	51498.67	5.19	-0.26	Plastid
Zm00001eb352310_P001	*ZmSET42*	460	1383	51526.04	8.48	-0.47	Cytoplasm
Zm00001eb362400_P001	*ZmSET43*	381	1146	43002.09	8.73	-0.52	Nucleus
Zm00001eb363830_P001	*ZmSET44*	696	2091	76860.6	8.19	-0.55	Nucleus
Zm00001eb406010_P001	*ZmSET45*	1081	3246	120702.33	7.51	-0.47	Nucleus
Zm00001eb413080_P001	*ZmSET46*	2181	6546	246601.86	6.72	-0.76	Nucleus
Zm00001eb413710_P001	*ZmSET47*	404	1215	43275.97	4.58	-0.28	Nucleus

Table. S2 Collinearity Analysis data

Chromosome	Gene	Start 1	End 1 (bp)	Chromosome	Gene	Start 1	End 1 (bp)

		(bp)				(bp)	
chr1	*ZmSET2*	49249772	49260050	chr9	*Zm00001eb396070_T002*	141317995	141332877
chr2	*ZmSET14*	181590171	181595315	chr4	*Zm00001eb178710_T001*	77609207	77612011
chr2	*ZmSET14*	181590171	181595315	chr7	*Zm00001eb310930_T001*	102661121	102664008
chr3	*ZmSET17*	3248587	3257024	chr8	*ZmSET40*	26004641	26032545
chr3	*ZmSET19*	159102896	159116690	chr8	*ZmSET44*	166463157	166477934
chr3	*ZmSET21*	218840069	218847282	chr8	*Zm00001eb359430_T003*	151550128	151559709
chr10	*ZmSET46*	58274786	58286152	chr4	*ZmSET22*	31285258	31298032
chr1	*ZmSET3*	206012653	206016982	chr4	*ZmSET23*	50769557	50774379

Table. S3 The numbers of introns and exons in the *ZmSET* gene family

Gene	exon	intron
ZmSET1	1	0
ZmSET2	16	15
ZmSET3	9	8
ZmSET4	11	10
ZmSET5	3	2
ZmSET6	15	14
ZmSET7	8	7
ZmSET8	17	16
ZmSET9	2	1
ZmSET10	8	7
ZmSET11	11	10
ZmSET12	2	1
ZmSET13	12	11
ZmSET14	3	2
ZmSET15	22	21
ZmSET16	4	3
ZmSET17	23	22
ZmSET18	1	0
ZmSET19	16	15
ZmSET20	3	2
ZmSET21	8	7
ZmSET22	21	20
ZmSET23	9	8
ZmSET24	11	10
ZmSET25	8	7
ZmSET26	12	11
ZmSET27	13	12
ZmSET28	5	4
ZmSET29	15	14
ZmSET30	3	2
ZmSET31	19	18
ZmSET32	12	11
ZmSET33	16	15
ZmSET34	4	3
ZmSET35	2	1
ZmSET36	26	25
ZmSET37	12	11
ZmSET38	6	5
ZmSET39	6	5
ZmSET40	23	22
ZmSET41	11	10
ZmSET42	7	6
ZmSET43	6	5
ZmSET44	16	15
ZmSET45	17	16
ZmSET46	21	20
ZmSET47	2	1

Table. S4 Synteny analyses data

	Chromosome	Gene	Chromosome	Gene
Zea mays vs Arabidopsis thaliana	Chr1	*ZmSET2*	Chr4	*AT4G02020.1*
	Chr8	*ZmSET40*	Chr3	*AT3G61740.1*
Zea mays vs Oryza sativa	Chr1	*ZmSET1*	Chr3	*Os03t0168700-00*
	Chr1	*ZmSET2*	Chr3	*Os03t0307800-01*
	Chr2	*ZmSET8*	Chr4	*Os04t0629100-01*
	Chr2	*ZmSET9*	Chr4	*Os04t0544100-01*
	Chr2	*ZmSET14*	Chr8	*Os08t0400200-00*
	Chr2	*ZmSET14*	Chr9	*Os09t0362900-01*
	Chr2	*ZmSET15*	Chr9	*Os09t0556700-01*
	Chr3	*ZmSET17*	Chr1	*Os01t0218800-01*
	Chr3	*ZmSET19*	Chr1	*Os01t0927000-01*
	Chr4	*ZmSET22*	Chr8	*Os08t0180100-01*
	Chr4	*ZmSET24*	Chr2	*Os02t0611300-01*
	Chr4	*ZmSET28*	Chr2	*Os02t0733800-01*
	Chr5	*ZmSET30*	Chr2	*Os02t0122700-00*
	Chr7	*ZmSET39*	Chr7	*Os07t0471100-01*
	Chr8	*ZmSET40*	Chr1	*Os01t0218800-01*
	Chr8	*ZmSET43*	Chr1	*Os01t0965500-01*
	Chr8	*ZmSET44*	Chr1	*Os01t0927000-01*

Table. S5 Primer list

Name	Upstream primer（5’-3’）	Downstream primer（3’-5’）
ZmSET3	AGTGATGATGTGGCTGGTGG	AGGTCTCGCTGCCAAATTCA
ZmSET9	GATGAGCGTCCAATGCCCTA	GTCTGAGCACCCACCTACAC
ZmSET12	ATCTCGGTTGGTGACGCTTT	AGTACGTCGCCATGATCGTC
ZmSET14	ACAGGAGCTGACCTACCACT	ACAGGAGCTGACCTACCACT
ZmSET28	ATTTTTCAGCGCCAGCCAAG	TCGGCAAGAAGCACCCTTAG
ZmSET32	GAAGGCCGACAAGACCATGA	GGGTGCTGAGATTTGGGGAA
ZmSET35	CAGTTGTTGATGCCAAGCCC	GGTCCAAATGGGGTCACACA
ZmSET39	GAAGCGGTCAGGTTTTGAGC	ACCAGAACAATCCTGCTCCG
ZmSET41	GCCCGAGGTGATTATGGAGA	TGTCTCTGGATCTGTTGACTCG
pCAMBIA1300-ZmSET9-HA	ACGAGCTCGGTACCCGGGATCCATGGAGACTGGGGCGGC(*BamHI*)	CGCGTACGAGATCTGGTCGACATACAGCCAGCCATCACACTCAA(Sal*I*)
HYG	CAAGACCAATGCGGAGC	ACAGGGTGTCACGTTGCAAG
AtACTIN	CTGGATTCTGGTGATGGTGTGTCT	GAACCACCGATCCAGACACTGTAC
AtABI5	ATGATCAAGAACCGCGAGTCTGC	CGGTTGTGCCCTTGACTTCAAAC
AtCAT1	TCCTGTTATCGTTCGTTTCTC	CAAAGTTCCCCTCTCTGGTGTA

Name	Upstream primer（5'-3'）	Downstream primer（3'-5'）
AtMYC2	ACGGATCAGGAGTACAGGAAAAA	GAAAAACCATTCCGTATCCGTC
AtRD22	TTTGGAATACGCGGGACACA	AAGGAACCATCGTGCAGCTT
ZmACTIN	TACGAGATGCCTGATGGTCAGGTCA	TGGAGTTGTACGTGGCCTCATGGAC
ZmSET9-BD	CATGGAGGCCGAATTCATGGAGACTGGGGCGG(*EcoRI)*	GCAGGTCGACGGATCCATACAGCCAGCCATCACACTCA(*BamHI*)
RBL-AD	GGAGGCCAGTGAATTCATGAACGTCCCTGTAGTCGATC(*EcoRI)*	CGAGCTCGATGGATCCGCTATCAATGTTAATCTCATCATCCTCAGTG(*BamHI*)
FIE1-AD	GGAGGCCAGTGAATTCATGCCGCCTTCCAAAGCA(*EcoRI)*	CGAGCTCGATGGATCCGGCGTCGGCGTCGGCG(*BamHI*)
FIE2-AD	GGAGGCCAGTGAATTCATGGCGAAGCTGGGCC(*EcoRI)*	CGAGCTCGATGGATCCGTTTCTGGAGCTCGGATGGTC(*BamHI*)

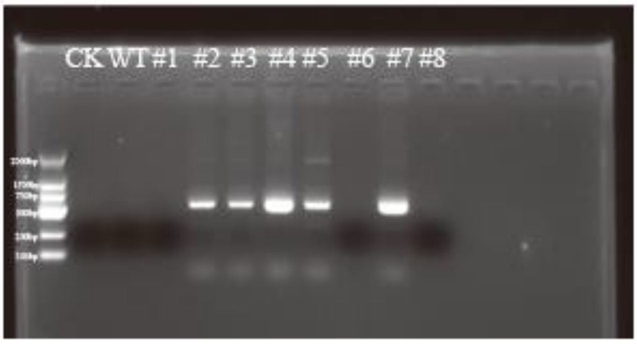

Figure S1. PCR identification of transgenic *Arabidopsis thaliana* lines. PCR amplification was performed using gene-specific primers to identify randomly selected putative transgenic *Arabidopsis thaliana* lines. M, DNA marker; CK, negative control (ddH_2O); WT, wild-type plants; #1–#8, eight randomly selected independent putative transgenic lines.

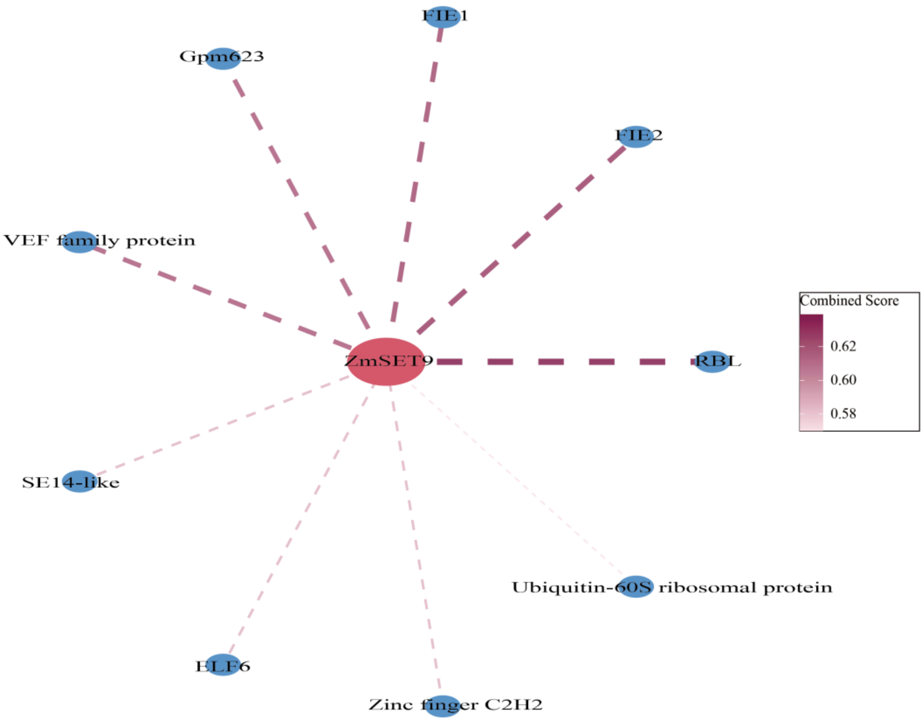

Figure S2. Protein-protein interaction network prediction of ZmSET9. Predicted interaction network of the ZmSET9 protein. The intensity of the red color positively correlates with the interaction score, with deeper red indicating a higher probability of interaction.
